# *Toxoplasma gondii* and *Neospora caninum* Antibodies in Dogs and Cats from Egypt and Risk Factor Analysis

**DOI:** 10.3390/pathogens11121464

**Published:** 2022-12-02

**Authors:** Dina B. Salama, Ragab M. Fereig, Hanan H. Abdelbaky, Moshera S. Shahat, Waleed M. Arafa, Shawky M. Aboelhadid, Adel E.A. Mohamed, Samy Metwally, Osama Abas, Xun Suo, Nishith Gupta, Caroline F. Frey

**Affiliations:** 1 Parasitology and Animal Disease Department, National Research Centre, Veterinary Research Institute, Giza 12622, Egypt; 2 National Animal Protozoa Laboratory, College of Veterinary Medicine, China Agricultural University, Beijing 100193, China; 3 Department of Biological Sciences, Birla Institute of Technology and Science, Pilani (BITS-P), Hyderabad 500078, India; 4 Department of Animal Medicine, Faculty of Veterinary Medicine, Division of Internal Medicine, South Valley University, Qena 83523, Egypt; 5 Veterinary Clinic, Veterinary Directorate, Qena 83511, Egypt; 6 Department of Animal Medicine, Faculty of Veterinary Medicine, South Valley University, Qena 83523, Egypt; 7 Parasitology Department, Faculty of Veterinary Medicine, Beni-Suef University, Beni-Suef 62511, Egypt; 8 Department of Animal Medicine, Faculty of Veterinary Medicine, Division of Clinical and Laboratory Diagnosis, South Valley University, Qena 83523, Egypt; 9 Department of Animal Medicine, Faculty of Veterinary Medicine, Division of Infectious Diseases, Damanhour University, Damanhour 22511, Egypt; 10 Department of Animal Medicine, Faculty of Veterinary Medicine, Division of Infectious Diseases, Alexandria University, Alexandria 21648, Egypt; 11 Department of Molecular Parasitology, Humboldt University, 10115 Berlin, Germany; 12 Department of Infectious Diseases and Pathobiology, Vetsuisse-Faculty, Institute of Parasitology, University of Bern, CH-3012 Bern, Switzerland

**Keywords:** Toxoplasmosis, Neosporosis, Canine, Feline, ELISA, odds ratio

## Abstract

Background: *Toxoplasma gondii* and *Neospora caninum* are major protozoan parasites of worldwide distribution and significance in veterinary medicine and, for *T. gondii*, in public health. Cats and dogs, as final hosts for *T. gondii* and *N. caninum,* respectively, have a key function in environmental contamination with oocysts and, thus, in parasite transmission. Very little is known about the prevalence of *T. gondii* infections in dogs and cats in Egypt, and even less about the prevalence of *N. caninum* in the same hosts. Methods: In the current study, 223 serum samples of both dogs (n = 172) and cats (n = 51) were investigated for specific antibodies to *T. gondii* and *N. caninum* using commercially available ELISAs. A risk factor analysis was conducted to identify factors associated with seropositivity. Results & discussion: Exposure to *T. gondii* was reported in 23.3% of the dogs and in 9.8% of the cats, respectively. In addition, *N. caninum*-specific antibodies were recorded in 5.8% of dogs and in 3.4% of cats. A mixed infection was found in two dogs (1.2%) and in one cat (2%). Antibodies to *T. gondii* in dogs were significantly more frequent in dogs aged 3 years or more and in male German Shepherds. As this breed is often used as watchdogs and was the most sampled breed in Alexandria governorate, the purpose “watchdog” (compared to “stray” or “companion”), the male sex, and the governorate “Alexandria” also had a significantly higher seroprevalence for *T. gondii*. No factors associated with antibodies to *N. caninum* could be identified in dogs, and no significant factors were determined in cats for either *T. gondii* or *N. caninum* infection. Our study substantially adds to the knowledge of *T. gondii* infection in dogs and cats and presents data on *N. caninum* infection in cats for the first and in dogs in Egypt for the second time.

## 1. Introduction

Domestic dogs and cats as companion animals are important for the mental health of the owners by reducing stress and anxiety [1], and dogs are also kept as watchdogs to protect properties. In Egypt, however, stray ones heavily outnumber owned dogs and cats. Both dogs and cats, especially stray ones, play a significant role in the transmission and epidemiology of many infectious diseases, including *Toxoplasma gondii* and *Neospora caninum* [2,3,4]. Cats and dogs, as final hosts for *T. gondii* and *N. caninum,* respectively, have a key function in environmental contamination with oocysts and, thus, in parasite transmission.

*Toxoplasma gondii* and *N. caninum* are closely related intracellular protozoan parasites displaying high phenotypic and genotypic similarities [5]. Toxoplasmosis, caused by *T. gondii*, is a global disease affecting almost all endothermic animals, including humans [6]. In human medicine and in the veterinary industry, mainly in small ruminants, *T. gondii* causes great harm in the case of prenatal transmission resulting in abortions, stillbirths, and neonatal fatalities. Neosporosis is caused by *N. caninum* and affects a large number of warm-blooded animals. It causes enormous economic losses in the cattle industry by inducing abortion [7,8].

Both parasites, *T. gondii* and *N. caninum* undergo sexual as well as asexual reproduction. Sexual reproduction occurs only in the definitive hosts, i.e., in felids for *T. gondii* [9] and in canids such as dog [10], coyote [11], dingo [12], and grey wolf [13] for *N. caninum*, respectively, and results in the shedding of oocysts. Asexual multiplication occurs in many tissues and organs of intermediate hosts, including Felidae and Canidae, and results in the formation of tissue cysts and potentially in clinical signs [14]. Infection thus occurs either orally by ingestion of oocysts, raw or semi-cooked meat containing tissue cysts, or vertically from an infected mother to the fetus.

Several reviews indicate that *T. gondii* infections are common in cats worldwide [6,15,16]. While mostly asymptomatic in cats, fatal systemic infections, usually including pneumonitis, may occur [14,15,17]. In dogs, clinical toxoplasmosis is rare and usually linked to immunosuppression [14]. Neuromuscular disorders caused by *T. gondii*, in one case identified as clonal Type 1, were described in two dogs [18,19]. In addition, toxoplasmosis was reported as severe and life-threatening respiratory distress in an immunosuppressed dog [20].

Clinical neosporosis in dogs is an important disease; it most commonly manifests as paralytic neuromuscular signs or as reproductive problems in infected bitches [21]. In cats, natural clinical infections with *N. caninum* have not been reported [7].

Few studies have investigated the seroprevalence of either *T. gondii* or *N. caninum* in dogs and cats in Egypt. Three studies assessed seroprevalence for *T. gondii* in cats, and results varied between 38.7% and 97.4% [22,23,24]. For *T. gondii* in dogs, three studies reported seroprevalence rates between 28% and 98.0% [3,25,26]. Only one study so far investigated 29 Egyptian dogs for anti-*N. caninum* antibodies and found a prevalence of 27.6% [27]. No data are available for the seroprevalence of *N. caninum* in cats in Egypt. Moreover, information on the seroprevalence of both parasites in cats and dogs is still missing for many Egyptian regions, and risk factor analysis has not been attempted so far. Thus, our study investigated the seroprevalence of *T. gondii* and *N. caninum* in dogs and cats from different governorates representing various regions of Egypt. In addition, various variables were assessed to identify factors associated with both infections in dogs and cats.

## 2. Materials and Methods

### 2.1. Ethical Statement

This study was performed according to standard procedures identified by the “Research Board” of the Faculty of Veterinary Medicine, South Valley University, Qena, Egypt. The study was approved by the Research Code of Ethics at South Valley University number 36 (RCOE-36). Blood samples were collected by highly trained veterinarians and after the verbal consent of the animal owners to participate in the study.

### 2.2. Animal Population and Location

**Figure 1:**
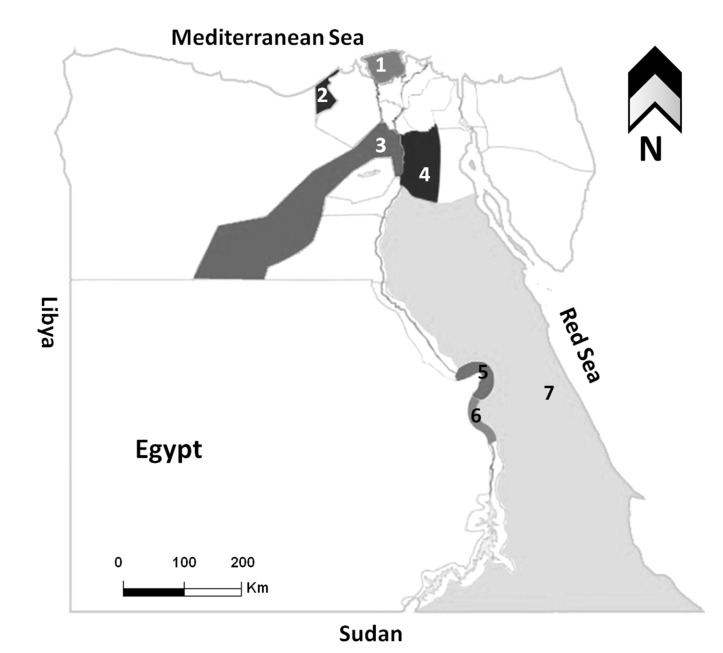
Map of Egypt showing the governorates where samples were collected. Areas with different colors refer to the investigated governorates, 1; Kafr Elsheikh, 2; Alexandria, 3; Giza, 4; Cairo, 5; Qena, 6; Luxor, 7; Red Sea.

A total of 223 blood samples were collected from both dogs (n = 172) and cats (n = 51) from different governorates representing most Egyptian geographic and climatic regions (Table 1, Figure 1). In the case of dogs, 100 blood samples were collected between January 2021 and March 2022, among which 50 dogs were referred to the pet clinic in Kafr Elsheikh for routine vaccinations or treatment of different clinical disorders (companion dogs), and 50 dogs were sampled at the dog shelter in Giza (stray dogs). In addition, a total of 31 samples were obtained from animal care centers at Luxor governorate (n = 13) and Hurghada city of Red Sea governorate (n = 23) during the period from May 2019 to March 2020 (stray dogs). Moreover, 36 dogs used as watchdogs for private properties were sampled from July to September 2022 from Alexandria. The age of the sampled dogs ranged from 3 months to 11 years, with a mean of 2.9 years. The female-to-male ratio was 78:94. Regarding cats, a total of 51 samples were collected in a period between January 2021 and September 2022 from pet animal clinics and hospitals in Cairo (n = 24), Kafr Elsheikh (n = 15), Qena (n = 4), and Red Sea (n = 8) governorates (companion cats). Except for dogs and cats referred to the animal clinics, all other tested animals were apparently and clinically healthy. The age of the sampled cats ranged from 8 months to 5 years, with a mean of 2.5 years. The female-to-male ratio was 27:24.

Variables such as age, sex, location, purpose (stray vs. companion vs. watchdog), and breeds were recorded for each sampled animal.

### 2.3. Sample Collection and Preparation

Blood samples were collected via puncture of the cephalic vein using glass tubes without anticoagulant, except for cats sampled in Cairo (n = 24), where EDTA blood was collected. All blood samples were kept in an icebox during transportation until separation, and serum and plasma samples, respectively, were stored at −20 °C until use.

### 2.4. ELISA Testing and Interpretation of Results

Serum samples from dogs and serum or plasma samples from cats were tested for anti-*T. gondii* and anti-*N. caninum* antibodies, respectively, using commercial Multi-species ELISA kits (ID Screen^®^ Toxoplasmosis Indirect Multi-species and ID Screen® *Neospora caninum* Competition, both ID Vet, Grables, France). Positive and negative control sera were provided in the kits, and the tests were performed following the manufacturer’s instructions. The optical density (OD) of ELISA results was read at 450 nm and measured with an Infinite^®^ F50/Robotic ELISA reader (Tecan Group Ltd., Männedorf, Switzerland).

The Toxoplasmosis kit detects specific immunoglobulin G (IgG) antibodies against the P30 *T. gondii* protein using a peroxidase-conjugated anti-multi-species secondary antibody. The percentage sample (*S*) to positive (*P*) ratio (*S/P* %) for each of the samples was calculated according to the following formula:

$$S⁄P\%\;\frac{\mathrm{OD}\;\mathrm{sample}-\mathrm{OD}\;\mathrm{negative}\;\mathrm{control}}{\mathrm{OD}\;\mathrm{positive}\;\mathrm{control}-\mathrm{OD}\;\mathrm{negative}\;\mathrm{control}\;}\times 100$$

Samples with *S*/*P*% values greater than 50% were considered to be positive, those between 40 and 50% were classified as doubtful, and measurements less than or equal to 40% were considered to be negative as per the manufacturer.

The *N. caninum* kit detects specific antibodies against a purified *N. caninum* extract using an anti-*N. caninum*peroxidase-conjugated competing antibody. The percentage sample (*S*) to negative (*N*) ratio (*S/N* %) for each of the test samples was calculated according to the following formula:

$$S⁄N\%\;\frac{\mathrm{OD}\;\mathrm{sample}}{\mathrm{OD}\;\mathrm{negative}\;\mathrm{control}\;}\times 100$$

Samples with *S*/*N*% values less than or equal to 50% were considered to be positive, those greater than 50% and less than or equal to 60% were classified as doubtful, and measurements greater than 60% were considered to be negative as per the manufacturer.

For both *T. gondii* and *N. caninum* test procedures, all samples were tested once except for the doubtful samples that were tested twice.

### 2.5. Statistical Analysis

The significance of the differences in the prevalence rates and risk factor analysis was assessed with Fisher exact probability test (two-tailed), 95% confidence intervals (including continuity correction), and odds ratios using an online statistical website www.vassarstats.net (accession dates; 15–17 April 2022) as described previously [28].

## 3. Results

Investigation of 172 dog sera revealed that 40 (23.3%) and 10 (5.8%) were seropositive for *T. gondii* and *N. caninum*, respectively (Table 2). Considering the different breeds investigated in the current study, the prevalence of *T. gondii* was 11/75 (14.7%) in native Baladi dogs, 21/47 (44.7%) in German Shepherds, 1/7 (14.3%) in Rottweiler*,* 1/5 (20%) in Doberman*,* 2/5 (40%) in a mixed breed, 1/7 (14.3%) in Pitbull*,* 1/4 (25%) in Belgian Malinois and Griffon Bruxellois, and 2/3 (66.7%) in Husky, respectively. In Boxer (n = 4)*,* Golden Retriever (n = 7), mixed Pitbull (n = 1), Great Dane (n = 1), and Labrador (n = 3), no antibodies to *T. gondii* were detected (Table 3). For *N. caninum* antibodies, results were 6/75 (8%) seropositive animals in Baladi, 1/7 (14.3%) in Rottweiler, 1/3 (33.3%) in Husky, 1/7 (14.3%) in Golden Retrievers, and 1/1 (100%) in Great Dane, while all other tested breeds were seronegative (Table 3).

In the case of the cat, an investigation of 51 samples revealed that 5 (9.8%) and 2 (3.9%) were seropositive for *T. gondii* and *N. caninum*, respectively (Table 2). Native Baladi cats, Persian, and Siamese cats were the most prevalent breeds in our study and among the cat population in Egypt. A seroprevalence of *T. gondii* of 0/12, 2/30 (6.7%), and 2/4 (50%) among Baladi, Persian, and Siamese cats, respectively, was demonstrated. Additionally, the only sampled British longhair tested positive for antibodies to *T. gondii* (100%). Antibodies to *N. caninum* were found in two cats only, one Baladi and one Siamese cat, respectively, resulting in a seroprevalence of 1/12 (8.3%) and 1/4 (25%) for each breed, respectively (Table 3). Regarding mixed infection, 2/172 dogs (1.2%) and 1/51 cats (2%) were demonstrated as seropositive for both *T. gondii* and *N. caninum* antibodies (Table 2).

Risk factor analysis was conducted to assess the influence of age, sex, purpose, geographical location, and breed on the seroprevalence of *T. gondii* in dogs and cats and *N. caninum* in dogs. All tested variables were identified as factors significantly associated with *T. gondii* infection in dogs (Table 4). Seroprevalence of *T. gondii* in dogs > 3 years old (40.4%) set as reference group was higher than that in 1–3 years old dogs (14.3%) (odds ratio [OR] = 0.2, *p* = 0.0009), and also than antibody level in younger dogs > 1 year old (20.7%) (OR = 0.4, *p* = 0.09). Moreover, the seropositive rate was significantly higher in male dogs (30.9%, OR = 2.7, *p* = 0.011) than in female ones (14.1%). Regarding breed, German Shepherd dogs exhibited a higher *T. gondii* seroprevalence (44.7%) than Baladi dogs (14.7%, OR = 0.2, *p* = 0.0006), or other breeds (16%, OR = 0.2, *p* = 0.0035) (Table 4).

When stray dogs as a reference (18.5%), a significantly higher seroprevalence was found in watchdogs (44.4%, OR = 3.5, *p* = 0.006), many of them German Shepherds (n = 30), but not for the companion dogs (16.4%, OR = 0.9, *p* = 0.821). Watchdogs were only sampled from Alexandria, and German Shepherds were overrepresented in the sample from this governorate. Thus, seroprevalence in Alexandria (44.4%, OR = 4.2, *p* = 0.007) was significantly higher than that in Giza (16%) set as reference. However, the differences were not significant in the case of Kafr Elsheikh (18%, OR = 1.2, *p* = 1), Luxor (30.8%, OR = 2.3, *p* = 0.249), or Red Sea (13%, OR = 0.8, *p* = 1), compared to Giza samples.

In order to analyze the effect of breed in more detail, three groups were created, namely native Baladi dogs (n = 75), German Shepherds (n = 47), and all other breeds (n = 50). In these smaller groups, age was no longer a significant risk factor for infection with *T. gondii* (Table 5). Male sex was retained as a risk factor in German Shepherds only (54.1%, OR = 10.6, *p* = 0.015) against female ones as a reference group (10%). Location and purpose were identified as associated factors in the “other breeds” group only, with companion dogs and dogs from Kafr Elsheikh having a lower seroprevalence (Table 5).

No risk factors were identified for *N. caninum* in dogs (Table S1) or for *T. gondii* in cats (Table S2). Risk factor analysis was not conducted for *N. caninum* in cats because of the low number of positive samples (2/43).

A summary of previous reports and their comparison with those in the current study on the seroprevalence of *T. gondii* and *N. caninum* in dogs and cats in Egypt is given in Table 6.

## 4. Discussion

In view of the paucity of available data for Egypt, the current study focused on the prevalence of *T. gondii* and *N. caninum* antibodies among dogs and cats. Very recent studies have demonstrated a high seroprevalence of *T. gondii* (46.1%) and a lower but still important seroprevalence of *N. caninum* (11.9%) in small ruminants in Egypt [29]. Moreover, cattle are affected by both parasites, with three in ten herds seropositive on bulk milk for *N. caninum* and one in ten seropositive for *T. gondii* [30]. Thus, the knowledge of the prevalence of these parasites in their final hosts is of utmost importance. In the present study, we used commercially available ELISAs to assess the seroprevalence in cats and dogs. The advantage of using commercial ELISAs in seroepidemiologic studies is that the results obtained should show little inter-laboratory variation and thus be widely comparable. However, this test also classified some samples as inconclusive, and it would have been nice to test them with a confirmatory test. Unfortunately, this was not possible in our study. Our study provides the first seroprevalence rate of *N. caninum* in cats in Egypt, and it was low at 3.4%. However, our study comprised companion cats only, and the sample size was quite small. Nevertheless, our study is one of the very few studies available on this topic worldwide and the first using the ID Screen *Neospora* ELISA in cats [7]. Therefore, we could not compare our results with other studies; neither could we shed light on the yet unknown risk factors for infection in cats [7]. Moreover, we were only the second group to investigate *N. caninum* antibodies in dogs in Egypt, after a lapse of about 20 years and on a considerably larger sample size. Compared with the earlier study, our seroprevalence of 5.8% was lower than the 27.6% obtained by El-Ghaysh et al. (2003) [27] using the direct agglutination test (DAT). However, our result was well in accordance with results obtained in other parts of the world using the same ID Screen *Neospora* ELISA: Dwinata et al. (2018) [31] obtained 3.4% in Indonesia, Villagra-Blanco et al. (2018) [32] 7.33% in Germany, and Lefkaditis et al. (2020) [33] 7.63% in Greece. It is interesting to see how little variation was detected in *N. caninum* seroprevalence in dogs when using the same method. A recent systematic review and meta-analysis of seroprevalence in the dog population worldwide established an overall seroprevalence of 23.31% in the Eastern Mediterranean region [34]. In this meta-analysis, the only significant associations with *N. caninum* infection in dogs were the continent, country, year, WHO regions, sample size, and diagnostic method used [34]. Consistently, no risk factors for *N. caninum* infection in dogs were observed in our study when analyzing the variables age, sex, breed, purpose, and governorate.

Few reports have investigated the seroprevalence of *T. gondii* in cats in Egypt, and information from Kafr Elsheikh and Cairo governorates has been entirely missing so far [22,23,24]. Our seropositive rate for *T. gondii* in cats (9.8%) was lower than that reported by Awad and Barakat (38.7%) (2019) [23] and Sherif et al. (54,2%) (2019) [24], both studies used rapid tests and Al-Kappany et al. (2010) [22] who reported 97.4% using a modified agglutination test (MAT) at a cut-off of 1:5, respectively. The ID Screen *Toxoplasma* ELISA we used was validated for use in cat serum and found to be equivalent to MAT with a cut-off of 1:40 [35]. We only found a few studies using the same ELISA in cats; these studies demonstrated a seroprevalence of 47% in owned cats in Romania [35] and of 21.93% in feral cats in Panama [36]. Our lower seroprevalence could thus be influenced by the test used, as well as by the studied population of sole companion cats. No risk factors were identified when analyzing age, sex, location, and breeds as predisposing factors for *T. gondii* infection in cats. This result was in concordance with Arruda et al. (2021) [37], who used IFAT, and referred to all tested variables. However, limited data on risk factors assessment of *T. gondii* infection in cats worldwide is available [15].

Arguably the most interesting results of our study concerned the seroprevalence of *T. gondii* in dogs in Egypt. Only three studies had investigated this topic, with a total of 176 dogs compared to the 172 included in our study, and focused on the greater Cairo region, namely Cairo and Giza governorates [3,25,26]. We now provided the first record for dogs of Kafr Elsheikh, Alexandria, and Luxor governorates. The seroprevalence of 23.3% for *T. gondii* in dogs obtained in our study was lower than the seroprevalences of 28%, 46.5%, and 98% reported by Khaled et al. (1982) [26], Rifaat et al. (1977) [25], and El Behairy et al. (2013) [3], respectively. These variations might be related to the different diagnostic tests used. The ID Screen *Toxoplasma* ELISA is also marketed for the use in dog sera and has been used for this purpose in studies from New Caledonia (Roqueplo et al., 2011; seroprevalence 32.8%) [38], from Grenada, West Indies (Sharma et al., 2014, seroprevalence 33.4%) [39], from the Philippines (Guy and Penuliar, 2016; seroprevalence 15.2%) [40], from Iran (Zarra-Nezhad et al., 2017; seroprevalence 46.67%) [41], from Panama (Fábrega et al., 2020; seroprevalence 25.70%) [36], and from Malaysia (Watanabe et al., 2020; seroprevalence 23.4%) [42]. Again, our results fit well within the rates obtained using the same diagnostic test.

In the risk factor analysis for *T. gondii* in dogs, we revealed that older and male dogs are more likely to be infected with *T. gondii* than younger and female dogs. In addition, location (Alexandria vs. other tested regions), purpose (watchdogs vs. stray or companion dogs), and breeds (German Shepherd vs. Baladi or other breeds) were identified as risk factors in our study. However, most of the dogs kept as watchdogs in our study were male German Shepherds, and they all originated from Alexandria, which might explain the high prevalence of *T. gondii* antibodies in these groups. Indeed, when the factors were analyzed within the German Shepherd group alone, only the male sex was retained as a risk factor for infection. However, our results were consistent with Raimundo et al. (2015) [43], who also found age and breed to be risk factors for infection, and with Arruda et al. (2021) [37], who reported breed as a risk factor. A study only including German Shepherds found a similar seroprevalence (46.3%) to ours in this breed [44]. The authors argued that this high seroprevalence might be related to the use of German Shepherd dogs as watchdogs in rural areas, guarding animal farms, or private properties, and thus have an increased risk of infection. This explanation also pertains to our study.

## 5. Conclusions

Our study provides valuable data on the prevalence of *N. caninum* and *T. gondii* among dogs and cats, adding substantially to the still scarce epidemiological knowledge on these important parasites in Egypt. We were able to include a large number of dogs in our study and to identify risk factors for their infection with *T. gondii*. As no successful and complete treatment or vaccine regimens are available to date to control both parasites, good animal husbandry practices, routine testing of the animals, and surveillance of the current epidemiological situation are recommended approaches for the prevention of infection.

## Figures and Tables

**Table 1:** Description of samples.

Region	Governorate	Dog	Sex (m:f)	Ownership /Purpose	Cat	Sex (m:f)	Ownership /Purpose	Total
Nile Delta	Kafr Elsheikh	50	29:21	Companion	15	9:6	owned	65
Western region	Alexandria	36	33:3	watchdog	-	-	-	36
Greater Cairo	Cairo	-	-		24	11:13	owned	24
	Giza	50	16:34	stray	-	-	-	50
Southern region	Qena	-	-		4	0:4	owned	4
	Luxor	13	4:9	stray	-	-	-	13
Eastern region	Red Sea	18 5	11:7 1:4	stray companion	8 -	4:4 -	owned -	26 5
Total	7 governorates	172	94:78		51	24:27		223

**Table 2:** Seroprevalence of *Toxoplasma gondii, Neospora caninum,* and mixed infection.

Type of Infection	Animal Species	No. of Tested	No. of Negative (%)	No. of Doubtful (%)	No. of Positive (%)	95% CI * Positive
*T. gondii*	Dog	172	117 (68)	15 (8.7)	40 (23.3)	17.3–30.4
	Cat	51	44 (86.3)	2 (3.9)	5 (9.8)	3.7–22.2
	Total	223	161 (72.2)	17 (7.6)	45 (20.2)	15.2–26.2
*N. caninum*	Dog	172	155 (90.1)	7 (4.1)	10 (5.8)	3–10.7
	Cat	51	49 (96.1)	0	2 (3.9)	0.7–14.6
	Total	223	204 (91.5)	7 (3.1)	12 (5.4)	3–9.4
Mixed infection	Dog	172	168 (97.7)	2 (1.2)	2 (1.2)	0.2–4.6
	Cat	51	50 (98)	0	1 (2)	0.1–11.8
	Total	223	218 (97.8)	2 (0.9)	3 (1.3)	0.4–4.2

* 95% CI calculated according to the method described by at http://vassarstats.net/, accessed date 15–17 April 2022.

**Table 3:** Seroprevalence of *Toxoplasma gondii* and *Neospora caninum* in dogs and cats in relation to breeds.

		*T. gondii*		*N. caninum*	
Species and Breed	No. Tested	No. Positive (%)	95% CI *	No. Positive (%)	95% CI
**Dog (n = 172)**					
Native Baladi	75	11 (14.7)	8–25.2	6 (8)	3.3–17.2
German Shepherd	47	21 (44.7)	30.5–59.8	0	0–9.4
Rottweiler	7	1 (14.3)	0.8–58.0	1 (14.3)	0.75–58
Doberman	5	1 (20)	1.1–70.1	0	0–53.7
Pitbull	7	1 (14.3)	0.8–58	0	0–43.9
Husky	3	2 (66.7)	12.5–98.2	1 (33.3)	1.8–87.5
Boxer	4	0	0–60.4	0	0–60.42
Griffon Bruxellois	4	1 (25)	1.3–78.1	0	0–60.4
Mixed breed	5	2 (40)	7.3–83	0	0–94.5
Belgian Malinois	4	1 (25)	1.3–78.1	0	0–60.4
Golden Retriever	7	0	0–43.9	1 (14.3)	0.8–58
Mixed pitbull	1	0	0–94.5	0	0–94.5
Labrador	3	0	0–69	0	0–69
Great Dane	1	0	0–94.5	1 (100)	5.5–100
**Cat (n = 51)**					
Native Baladi	12	0	0–30.1	1 (8.3)	0.4–40.2
Persian	30	2 (6.7)	1.2–23.5	0	0–14.1
Siamese	4	2 (50)	9.2–90.8	1 (25)	1.3–78.1
Turkish Angora	2	0	0–80.2	0	0–80.2
British Longhair	1	1 (100)	5.5–100	0	0–94.5
Mixed breed	2	0	0–80.2	0	0–80.2

* 95% CI calculated according to the method described by (http://vassarstats.net/, accessed date 15–17 April 2022).

**Table 4:** Risk factors for *Toxoplasma gondii* antibodies in dogs.

Analyzed Factor	No. of Tested	No. of Negative (%)	No. of Positive (%)	OR (95% CI) #	*p-*Value *
**Age**					
<1 year	29	23 (79.3)	6 (20.7)	0.4 (0.1–1.1)	0.09
1–3 years	91	78 (85.7)	13 (14.3)	0.2 (0.1–0.6)	0.0009
>3 years	52	31 (69.6)	21 (40.4)	Ref.	Ref.
**Sex**					
Female	78	67 (85.9)	11 (14.1)	Ref.	Ref.
Male	94	65 (69.1)	29 (30.9)	2.7 (1.3–5.9)	0.011
**Location**					
Kafr Elsheikh	50	41 (82)	9 (18)	1.2 (0.4–3.3)	1
Giza	50	42 (84)	8 (16)	Ref.	Ref.
Alexandria	36	20 (55.6)	16 (44.4)	4.2 (1.5–11.4)	0.007
Luxor	13	9 (69.23)	4 (30.8)	2.3 (0.6–9.5)	0.249
Red Sea	23	20 (87)	3 (13)	0.8 (0.2–3.3)	1
**Ownership status**					
Stray	81	66 (81.5)	15 (18.5)	Ref.	Ref.
Companion	55	46 (83.6)	9 (16.4)	0.9 (0.3–2.1)	0.821
Watchdog	36	20 (55.6)	16 (44.4)	3.5 (1.5–8.4)	0.006
**Breeds**					
Native Baladi	75	64 (85.3)	11 (14.7)	0.2 (0.1–0.5)	0.0006
German Shepherd	47	26 (55.3)	21 (44.7)	Ref.	Ref.
Others	50	42 (84)	8 (16)	0.2 (0.1–0.6)	0.0035

# Odds ratio at 95% confidence interval as calculated by http://vassarstats.net/ (access time, 15–17 April 2022). * p value was evaluated by Fisher’s exact probability test (two-tailed). Ref.; value used as a reference.

**Table 5:** Risk factors assessment of *Toxoplasma gondii* infection in dogs in relation to breeds.

Variables	Native Baladi Dogs (n = 75)					German Shepherd (n = 47)					Other Breeds (n = 50)				
	No. Tested	No. Negative (%)	No. Positive (%)	OR (95% CI) #	*p*-Value *	No. Tested	No. Negative (%)	No. Positive (%)	OR (95% CI) #	*p*-Value *	No. Tested	No. Negative (%)	No. Positive (%)	OR (95% CI) #	*p*-Value *
**Age**															
<1 year	18	14 (77.8)	4 (22.2)	1.4 (0.2–9.4)	1	2	1 (50)	1 (50)	0.9 (0.05–15.3)	1	9	8 (88.9)	1 (11.1)	0.2 (0.04–1.3)	0.165
1–3 years	45	40 (88.9)	5 (11.1)	0.6 (0.1–3.7)	0.63	17	12 (70.6)	5 (29.4)	0.4 (0.1–1.3)	0.135	29	26 (89.7)	3 (10.3)	0.3 (0.02–2.8)	0.338
>3 years	12	10 (83.3)	2 (16.7)	Ref.	Ref.	28	13 (46.4)	15 (53.6)	Ref.	Ref.	12	8 (66.7)	4 (33.3)	Ref.	Ref.
**Sex**															
Male	27	21 (77.8)	6 (22.2)	2.5 (0.7–9)	0.188	37	17 (45.9)	20 (54.1)	10.6 (1.2–92.3)	**0.015**	30	27 (90)	3 (10)	0.3 (0.07–1.6)	0.24
Female	48	43 (89.6)	5 (10.4)	Ref.	Ref.	10	9 (90)	1 (10)	Ref.	Ref.	20	15 (75)	5 (25)	Ref.	Ref.
**Ownership**															
Stray	69	58 (84.1)	11 (15.9)	Ref.	Ref.	3	2 (66.7)	1 (33.3)	Ref.	Ref.	9	5 (55.6)	4 (44.4)	Ref.	Ref.
Companion	6	6 (100)	0	0.4 (0.02–7.4)	0.538	14	9 (64.3)	5 (35.7)	1.1 (0.08–15.5)	1	35	31 (88.6)	4 (11.4)	0.2 (0.03–0.9)	**0.042**
Watchdog	0	-	-	-	-	30	15 (50)	15 (50)	2 (0.2–24.5)	1	6	5 (83.3)	1 (16.7)	0.3 (0.02–3.1)	0.58
**Location**															
Kafr Elsheikh	0	-	-	-	-	14	9 (64.3)	5 (35.7)_	Ref.	Ref.	36	32 (88.9)	4 (11.1)	0.04 (0.003–0.5)	**0.013**
Giza	45	40 (88.8)	5 (11.1)	Ref.	Ref.	1	1 (100)	0	0.6 (0.02–16.7)	1	4	1 (25)	3 (75)	Ref.	Ref.
Alexandria	0	-	-	-	-	30	15 (50)	15 (50)	1.8 (0.5–6.7)	0.519	6	5 (83.3)	1 (16.7)	0.07 (0.003–1.5)	0.19
Luxor	6	4 (66.7)	2 (33.3)	4 (0.6–27.7)	0.186	2	1 (50)	1 (50)	1.8 (0.1–35.5)	1	5	4 (80)	1 (20)	0.08 (0.004–1.9)	0.206
Red Sea	23	20 (87)	3 (23)	1.2 (0.3–5.5)	1	0	-	-	-	-	0	-	-	-	-

# Odds ratio at 95% confidence interval as calculated by http://vassarstats.net/ (accessed date 15–17 April 2022). * p value was evaluated by Fisher’s exact probability test (two-tailed). Ref.; value used as a reference. (-) Not calculated variables because of no samples.

**Table 6:** Previous reports on the prevalence of *Toxoplasma gondii* and *Neospora caninum* in dogs and cats in Egypt.

Protozoa	Host *	Governorate	No. Tested	No. Positive (%)	Test	Reference
*T. gondii*	Stray, companion & Watchdogs	5 Governorates	172	40 (23.3)	Indirect ELISA	Current study
*T. gondii*	Owned cat	4 Governorates	51	5 (9.8)	Indirect ELISA	Current study
*T. gondii*	Household cats	Giza	212	82 (38.67)	RCIA *	[23]
*T. gondii*	Stray and household cats	Giza, Minofyia, Red Sea	240	IgM; 35% & 33.33%, IgG; 55% & 53.33% in stray & household cats, respectively	On site rapid test	[24]
*T. gondii*	Feral cats	Giza	158	154 (97.4)	MAT *	[22]
*T. gondii*	Dogs	Cairo	82	38 (46.5)	Dye test *	[25]
*T. gondii*	Stray dogs	Cairo	43	12 (28)	Dye test	[26]
*T. gondii*	Stray dogs	Giza	51	50 ( 98.0)	MAT	[3]
*N. caninum*	Stray dogs	Unknown/Egypt	29	8 (27.6)	DAT	[27]
*N. caninum*	Stray, companion & watchdogs	5 Governorates	172	10 (5.8)	Competitive ELISA	Current study
*N. caninum*	Owned cat	4 Governorates	51	2 (3.9)	Competitive ELISA	Current study

* Host name was used as referred by the original paper. RCIA; Rapid chromatographic immune assay, MAT; modified agglutination test, DAT; direct agglutination test.

## Data Availability

All data generated and analyzed during this study are included in this published article. Raw data supporting the findings of this study are available from the corresponding author at the request.

## Supplementary Materials

The following supporting information can be downloaded at: https://www.mdpi.com/article/10.3390/pathogens11121464/s1, Table S1: Risk factors for *N. caninum* antibodies in dogs. Table S2: Risk factors for *T. gondii* antibodies in cats.
