# Supplementary material for: Toxoplasma gondii and Neospora caninum Antibodies in Dogs and Cats from Egypt and Risk Factor Analysis
Source: Pathogens. 2022 Dec 2;11(12):1464. doi: 10.3390/pathogens11121464 (PMC9785966; doi:10.3390/pathogens11121464)
Supplement: Supplementary file 1 [file pathogens-11-01464-s001.zip › pathogens-2064508-Supplementary.pdf]

Supplementary Materials

Table S1. Risk factors for *N. caninum* antibodies in dogs

Analyzed factor	No. of Tested	No. of negative (%)	No. of positive (%)	OR (95% CI) *	*p*-value #
**Age**					
< 1 year	29	28 (96.6)	1 (3.4)	Ref	Ref
1–3 years	91	83 (91.2)	8 (8.8)	2.7 (0.3–22.6)	0.686
> 3 years	52	51 (98.1)	1 (1.9)	0.5 (0.03–9.1)	1.00
**Sex**					
Female	78	71 (91)	7 (9)	Ref	Ref
Male	94	91 (96.8)	3 (3.2)	0.3 (0.08–1.3)	0.188
**Location**					
Kafr Elsheikh	50	46 (92)	4 (8)	1 (0.2–4.2)	1
Giza	50	46 (92)	4 (8)	Ref	Ref
Alexandria	36	36 (100)	0	0.1 (0.007–2.7)	0.136
Luxor	13	12 (92.3)	1 (7.7)	1 (0.1–9.3)	1.00
Red Sea	23	22 (95.7)	1 (4.3)	0.5 (0.06–5)	1.00
**Ownership status**					
Stray	81	76 (93.8)	5 (6.2)	Ref	Ref
Companion	55	50 (92)	5 (8)	1.5 (0.4–5.5)	0.525
Watchdog	36	36 (100)	0	0.2 (0.01–3.5)	0.321
**Breeds**					
Native Baladi	75	70 (93.3)	5 (6.7)	Ref	Ref
Others	97	93 (95.9)	4 (4.1)	0.6 (0.2–2.3)	0.505

# Odds ratio at 95% confidence interval as calculated by http://vassarstats.net/.

* *p* value was evaluated by Fisher exact probability test (two-tailed).

Ref.; value used as a reference.

Table S2. Risk factors for *T. gondii* antibodies in cats.

Analyzed factor	No. of Tested	No. of negative (%)	No. of positive (%)	OR (95% CI) *	*p*-value #
**Age**					
≤ 2 year	31	30 (96.8)	1 (3.2)	Ref	Ref
> 2 years	20	16 (80)	4 (20)	7.5 (0.8–72.9)	0.071
**Sex**					
Female	27	24 (88.9)	3 (11.1)	Ref	Ref
Male	24	22 (91.7)	2 (8.3)	1.35 (0.20–9.02)	1.00
**Location**					
Kafr Elsheikh	15	12 (80)	3 (20)	Ref	Ref
Cairo	24	22 (91.7)	2 (8.3)	0.7 (0.1–4.8)	0.35
Qena	4	4 (100)	0	0.8 (0.03–17.8)	1.00
Red Sea	8	8 (100)	0	0.4 (0.02–8.8)	1.00
**Breeds**					
Persian	33	31 (93.9)	2 (6.1)	Ref	Ref
Siamese	6	4 (66.7)	2 (33.3)	7.8 (0.8–71.3)	0.104
Others	12	11 (91.7)	1 (8.3)	1.4 (0.1–17.3)	1.00

# Odds ratio at 95% confidence interval as calculated by http://vassarstats.net/.

* *p* value was evaluated by Fisher exact probability test (two-tailed).

Ref.; value used as a reference.
